# Predict Score: A New Biological and Clinical Tool to Help Predict Risk of Intensive Care Transfer for COVID-19 Patients

**DOI:** 10.3390/biomedicines9050566

**Published:** 2021-05-18

**Authors:** Mickael Gette, Sara Fernandes, Marion Marlinge, Marine Duranjou, Wijayanto Adi, Maelle Dambo, Pierre Simeone, Pierre Michelet, Nicolas Bruder, Regis Guieu, Julien Fromonot

**Affiliations:** 1 Laboratory of Biochemistry, Timone University Hospital, APHM, 13005 Marseille, France or mickaelgette@gmail.com (M.G.); marion.marlinge@ap-hm.fr (M.M.); marine.duranjou@ap-hm.fr (M.D.); maylis.adi-wijayanto@ap-hm.fr (W.A.); maelle.dambo@ap-hm.fr (M.D.); julien.fromonot@univ-amu.fr (J.F.); 2 Center for Research and Studies on Health Services and Quality of Life, Aix-Marseille University, 13005 Marseille, France; sarah.fernandes@etu.univ-amu.fr; 3 INSERM, INRAE, C2VN, Aix-Marseille University, 13005 Marseille, France; pierre.michelet@univ-amu.fr; 4 Department of Anesthesiology and Intensive Care, Timone University Hospital, Aix Marseille University APHM, 13005 Marseille, France; pierre.simeone@ap-hm.fr (P.S.); nicolas.bruder@ap-hm.fr (N.B.); 5 Department of Emergency Medicine and Intensive Care, Timone University Hospital, APHM, 13005 Marseille, France

**Keywords:** COVID-19, score, biology, intensive care

## Abstract

Background: The COVID-19 crisis has strained world health care systems. This study aimed to develop an innovative prediction score using clinical and biological parameters (PREDICT score) to anticipate the need of intensive care of COVID-19 patients already hospitalized in standard medical units. Methods: PREDICT score was based on a training cohort and a validation cohort retrospectively recruited in 2020 in the Marseille University Hospital. Multivariate analyses were performed, including clinical, and biological parameters, comparing a baseline group composed of COVID-19 patients exclusively treated in standard medical units to COVID-19 patients that needed intensive care during their hospitalization. Results: Independent variables included in the PREDICT score were: age, Body Mass Index, Respiratory Rate, oxygen saturation, C-reactive protein, neutrophil–lymphocyte ratio and lactate dehydrogenase. The PREDICT score was able to correctly identify more than 83% of patients that needed intensive care after at least 1 day of standard medical hospitalization. Conclusions: The PREDICT score is a powerful tool for anticipating the intensive care need for COVID-19 patients already hospitalized in a standard medical unit. It shows limitations for patients who immediately need intensive care, but it draws attention to patients who have an important risk of needing intensive care after at least one day of hospitalization.

## 1. Introduction

In December 2019, medical teams of Wuhan, Hubei, China discovered a novel coronavirus responsible for acute respiratory distress syndrome (ARDS). They were able to identify this new pathogen using next-generation sequencing and transforming the real-time polymerase chain reaction (RT-PCR) in a “user-friendly” diagnostic tool for laboratories with little familiarity with this technology [1].

The severe acute respiratory syndrome coronavirus 2 propagated around the world until it was declared a Worldwide Public Health Emergency on the 30th of January 2020 by the World Health Organization (WHO), being considered a threat to health care systems [2]. The WHO emergency committee recommended massive detection strategies, isolation of contaminated patients, early treatment and new technological contact-tracing systems to limit the spread of COVID-19. However, several months after the beginning of this pandemic, two facts remain constant: the lack of resources, and that isolation has been the only effective strategy in limiting the spread of the disease.

Hospitals had to adapt to this new situation daily, restricting their access to non-urgent diseases, increasing the number of beds in their intensive care unit (ICU), and isolating COVID patients despite the lack of adequate protective equipment (qualitatively and quantitatively) for health workers and non-COVID patients [3].

In France, 89,818 patients were hospitalized, of which 4387 required intensive care and 23,686 died from COVID between 1 March 2020 and 28 April 2021 [4]. In the Provence Alpes Cote d’Azur Region, during the same period, 1220 patients were hospitalized, of which 295 needed intensive care treatment, and 82 died from COVID [5].

Modern medical biology has the potential to acquire an important role in this type of crisis, as it is indispensable for diagnosis and useful for the development of a treatment plan and guiding medical decisions and hospitalization scheduling [6,7,8]. Previous studies have identified biomarkers that significantly document a high risk of progression to severe forms of COVID-19 [9], such as interleukin-6 and D-Dimer levels. Others have proposed the use of a composite risk score [10,11,12], using clinical data similarly to the National Early Warning Score 2 (NEWS 2), medical history and different biomarkers, but requiring a web calculator.

The aim of this study was to create a composite risk score using biological and clinical parameters, that evaluated the risk of COVID-19-positive patients hospitalized in a Standard Medical Unit (SMU) needing intensive care during the days following hospitalization. Thus, helping medical teams anticipate the level of medical care a patient will need, and therefore allowing them to use their resources wisely, particularly ICU beds and artificial respirators. During this study, the main preoccupation was to build a user-friendly score, using biological parameters which are widely available throughout the world, easily measured clinical parameters and the patient’s intrinsic constants, without neglecting discrimination capacity.

## 2. Materials and Methods

### 2.1. Study Design and Patient Selection

We designed a retrospective monocentric study, including all health care centers of the Assistance Publique des Hôpitaux de Marseille (AP-HM) (Public Assistance of Marseille Hospitals), France. Biological resources, medical imaging and clinical records were all produced in different AP-HM sites.

**Figure 1:**
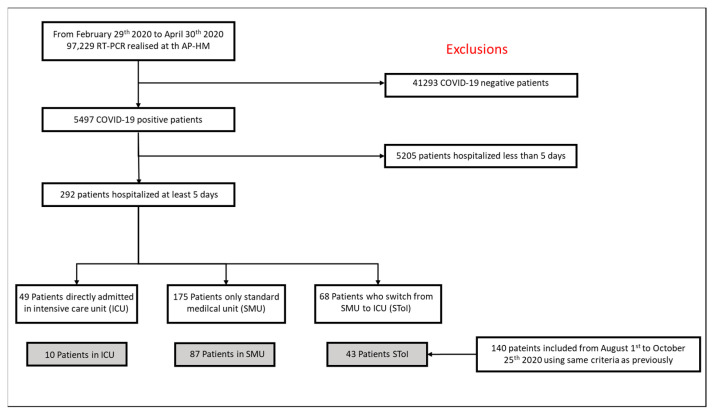
Flow chart.

From 29 February 2020 to 30 April 2020, all adult patients diagnosed with COVID-19 according to WHO guidelines [13] were initially included in a first cohort (see Figure 1). Patient selection did not consider patients’ characteristics, age, sex, medical history, treatments, or initial clinical evaluation and vital signs. This first cohort was used to construct the PREDICT score (predicting risk factors for early determination of ICU transfer). A second cohort of patients was enrolled, from 1 August to 25 October 2020, using the same criteria as previously, to validate the score.

The subjects were separated into three different groups, based on disease severity and their requirement for intensive care:

1. Patients admitted to the Standard Medical Unit were included in the SMU group;
2. Patients admitted directly into the Intensive Care Unit directly were included in the ICU group;
3. Patients that initially were admitted to the Standard Medical Unit for at least 24 h, but subsequently needed to be transferred to the Intensive Care Unit were included in a third group, named Standard to Intensive Care (STol) group.

Two reasons motivated this choice: firstly the need for intensive care is associated with complications which are unmanageable by a standard medical unit (SMU), secondly the limited number of places in Intensive Care Unit (ICU) confers a critical value to their management. During their practice, physicians employed general severity tools for respiratory diseases and used their clinical judgement to decide when patients needed intensive care unit [14,15], as stated in international guidelines and recent recommendations, but no specific scores.

In the training cohort, 175 patients were admitted to standard medical unit (SMU group), 49 patients were admitted to the intensive care unit directly (ICU group) and 68 patients were initially admitted in a standard medical unit but later required intensive care (STol group). In the validation cohort, 87 were included in the SMU group, 10 patients in the ICU group, and 43 patients in the SToI group.

To train the PREDICT score, after having analyzed a lot of parameters (intrinsic, comorbidity, vital sign, biologic) and a lot of combinations of these, a multivariate analysis highlighted a significative combination of age, Body Mass Index, oxygen saturation (SpO_2_) at admission, respiratory rate at admission, neutrophil–lymphocyte ratio, C-reactive protein and lactate dehydrogenase.

### 2.2. Exclusion Criteria

Patients with fewer than 5 days of hospitalization were excluded, to be coherent with virologic load following, given that previous studies reported that the median time of RT-PCR ending detection was 10 days [16] and, in the two cohorts, median time between first symptoms and hospitalization was 5 days.

Furthermore, patients who stayed less than 5 days in hospital were suffering from a low-severity form of COVID-19 and are beyond the scope of this study.

### 2.3. Clinical, Imaging and Laboratory Data Collection

Axigate software was used to collect clinical data from medical records, like vital sign monitoring (body temperature [T °C], cardiac and respiratory frequency, oxygen saturation [SpO_2_], systolic and diastolic blood pressure), symptomatology at admission (fever, dyspnea, cough, anosmia, ageusia, digestive troubles), oxygen requirement, height, weight, body-mass index, and past medical history. Oxygen saturation (SpO_2)_ was evaluated before oxygen therapy in all cases.

Furthermore, important dates were recorded, such as the day of symptom commencement, first day of hospitalization, changeover date to ICU if required, and release date from ICU. Further, medical progress notes were collected, and important features such as endotracheal intubation and acute respiratory distress symptoms were recorded. Regarding imaging, radiologic reports of unenhanced low-dose chest-computed tomography were used; the AP-HM imaging unit uses a standardized report with qualitative appreciation lung damage sorted into four levels: Absence, Minor, Intermediary, Severe. Finally, laboratory data were collected with the Nextlab Software used by both AP-HM laboratories.

### 2.4. Laboratory Findings

Based on previous studies and considering our aim to use only common parameters, we decided to collect data on natremia (Na), C-reactive protein (CRP), ferritinemia (FRT), lactate dehydrogenase (LDH), creatinine (CREAT), total bilirubin (BILI), aspartate aminotransferase (ASAT), and alanine aminotransferase (ALAT). Biochemical parameters were measured with a COBAS C701 provided by Roche Hitachi, and all reagents used came from Roche (Meylan, France).

Additionally, lymphocyte count (LY) and neutrophils cells count (NEU) were recorded to use the neutrophil–lymphocyte ratio (NLR), as a significant biomarker; platelet count was also included. These analyses were performed by a XN–3000 provided by Sysmex. D-Dimer and fibrinogen measures were also recorded, performed on a Star Max provided by Stago; reagents were provided by Stago as well (Stago Canada, Ltée).

A raw laboratory parameter database was created to record this information, allowing for kinetic-follow up of each parameter for each patient.

### 2.5. Definitions

To evaluate the clinical severity at admission, the NEWS 2 was used, which includes heart and respiration rate, oxygen saturation and supplementation, systolic blood pressure, consciousness, temperature and age [17]. It is an easy way to classify patient severity, helping medical teams treat their patients correctly. Further, age has been reported as an independent risk factor for disease severity [18,19], with a threshold at 65 years. Radiological severity was defined as Minor when patients had 3 compromised sites, with 3 lobules affected on each site (maximum 9 lobules); Intermediary, when patients had a minimum of 10 lobules affected, but less than 50% of total segmental volume; Severe, when more than 50% of total segmental volume was affected. Acute respiratory failure (ARF) was defined as respiratory rate > 20 (or accessory muscle use for ventilation), and hypoxemia (oxygen partial pressure (PaO2) lower than 60 mm Hg on breathing room air), acute respiratory distress syndrome (ARDS, Berlin definition); acute respiratory failure not explained by cardiac failure or fluid overload with bilateral lung opacities on chest imaging and PaO2/FiO2 < 300 with positive end-expiratory pressure > 5 cm H2O (Fraction of inspired oxygen: FiO2). [20].

### 2.6. Statistical Analysis

Two cohorts were analyzed: the training cohort (N = 292) and the validation cohort (n = 140). The baseline patient characteristics were expressed as frequencies and percentages for categorical variables and as mean ± standard deviation or as median and interquartile ranges for continuous variables. First, three comparisons were performed between groups: SMU vs. ICU, SMU vs. StoI, and ICU vs. StoI. The Shapiro–Wilk test was applied to assess the normality of the data. Continuous variables were compared using Mann–Whitney U-test; categorical variables were compared using the Chi-square test or Fisher’s exact test, as appropriate. The comparisons were performed between groups within each cohort.

Second, to compare the kinetics of the biological parameters over time between the three groups, we performed separate linear mixed model (LMM) analysis for 14 biological parameters collected at different times. We also performed univariate logistic regressions to identify which clinical parameters were significantly associated with the likelihood of being transferred to an intensive care unit. For easier application to the prediction score model, significant continuous parameters were then converted to categorical variables according to the optimal cutoff value derived from the Youden index (C-reactive protein (CRP), lactate dehydrogenase (LDH), neutrophil–lymphocyte ratio (NLR), peripheral oxygen saturation (SpO_2_) and respiratory rate). Body mass index ≥ 30 kg/m^2^ and age < 75 years were identified as risk factors for ICU transfer. The covariates included were: time, temperature, SpO_2_, Respiratory rate, age, Body Mass Index, sex and comorbidities (diabetes, hypertension, cardio-vascular diseases, dyslipidemia, chronic obstructive pulmonary disease, asthma, tobacco, active and remission cancer, kidney disease).

Third, a score to predict the need for transfer to an intensive care unit was constructed using the training cohort by performing a multivariate logistic regression analysis. The dependent variable was transfer to ICU (yes–no); eigh independent variables (age, body mass index, respiratory rate, oxygen saturation, neutrophil–lymphocyte ratio at admission and in follow-up, CRP in follow-up, LDH in follow-up, and time) were entered in the model. The multivariate regression coefficients were used to assign integer points for the prediction score; each coefficient was multiplied by two and rounded to the nearest integer. Individual risk estimates were based on the sum of weighted scores for each variable; the in-hospital time was time-weighted to identify patients at low risk of being transferred to an intensive care unit. Results were presented as odd ratios and their 95% confidence intervals (CIs). The PREDICT score was subsequently tested on the validation cohort.

Fourth, the PREDICT score was calculated at three different times: admission, day 1, and day 2. For each score, area under the receiver operating characteristic (ROC) curves and the Youden index were calculated. Youden index is defined for all point of ROC curves (sensitivity + specificity − 1) and the maximum value of this index was selected to be the optimal cut-off point and name Youden’s threshold. Sensitivity, specificity, positive predictive values, and negative predictive values were provided as percentages and their respective 95% CIs.

Fifth, the biological parameters were compared between the groups: at each time (Student’s *t*-test or Mann–Whitney test) and globally, on the different evaluation times (generalized linear models). A *p* value < 0.05 was considered statistically significant. All statistical analyses were performed using SPSS version 20.0 (IBM, Armonk, NY, USA).

Informed consent was obtained from all subjects involved in the study.

## 3. Results

### 3.1. Patient Characteristics

Univariate analysis between SMU vs ICU groups and SMU vs STol groups, identified that patients with ages inferior to 75 years were more likely to be admitted to ICU (Odd Ratio 2.3 (IC 95%: 1.03–5.1; *p* = 0.0481) and Odd Ratio 2.3 (IC 95%: 1.2–4.3; *p* = 0.005), respectively). Table 1, Table 2, Table 3 and Table 4. Table 5 (and Table S1) represents the method calculation of the NEWS2 score.

This parameter is still significative in a multivariate analysis, including body mass index, respiratory rate, SpO_2_, neutrophil–lymphocyte ratio, C-reactive protein and lactate dehydrogenase comparing SMU vs (ICU + SToI) groups (OR 231.2; 95% CI: [8.1–,611.4]; *p* = 0.001]) (Table 6). All multivariate analysis always used same parameters (age, body mass index, respiratory rate, SpO_2_, neutrophil–lymphocyte ratio, C-reactive protein and lactate dehydrogenase).

Moreover, body mass index superior or equal to 30 has already been observed [21] as key comorbid factor in the intensive care units. In this study, the percentage of subjects with body mass index superior or equal to 30 was 22.9%, 22.1% and 49% in the SMU, SToI and ICU groups, respectively, representing a significative difference. Multivariate analysis for this criterion also showed statistical significance, with an odds ratio 96.4 (95% CI: [4.8–1928.1]; *p* = 0.003), patients with a body mass index superior to 30 had greater risk of needing intensive care unit treatment (Table 6).

However, only parameters identified as independent risk factors through multivariate regression analysis were used to build the score: age, body mass index, respiratory rate, and SpO_2_ (Table 1 and Table 2). The same analysis and results are presented in Table 3 and Table 4 for the validation cohort.

### 3.2. Patient Vital Signs

Notable differences appeared after analyzing differences between groups on easily measurable vital signs (respiratory rate, temperature, SpO_2_) (Table 1 and Table 3).

Vital parameters for SMU, SToI and ICU groups were: respiratory rate (Median: 22, IQR: 18–26), (Median: 24, IQR: 20–30), and (Median: 30, IQR: 25–35), respectively; body temperature (Median: 37.1, IQR: 36.7–38), (Median: 37.9, IQR: 37–38.5), and (Median: 38.1, IQR: 37.1–38.8), respectively; SpO_2_ (Median: 96, IQR: 93–97), (Median: 95, IQR: 93–96), (Median: 94, IQR: 89–95), respectively.

Multivariate analysis for those criteria showed a statistical significance (OR 348.6 (IC 95%: 10.5–11,567.9; *p* = 0.001) and OR 244.6 (IC 95%: 9.2–6,490.1; *p* = 0.005), respectively, for respiratory rate and SpO_2_) (Table 6).

### 3.3. Patient Biological Parameters

**Figure 2:**
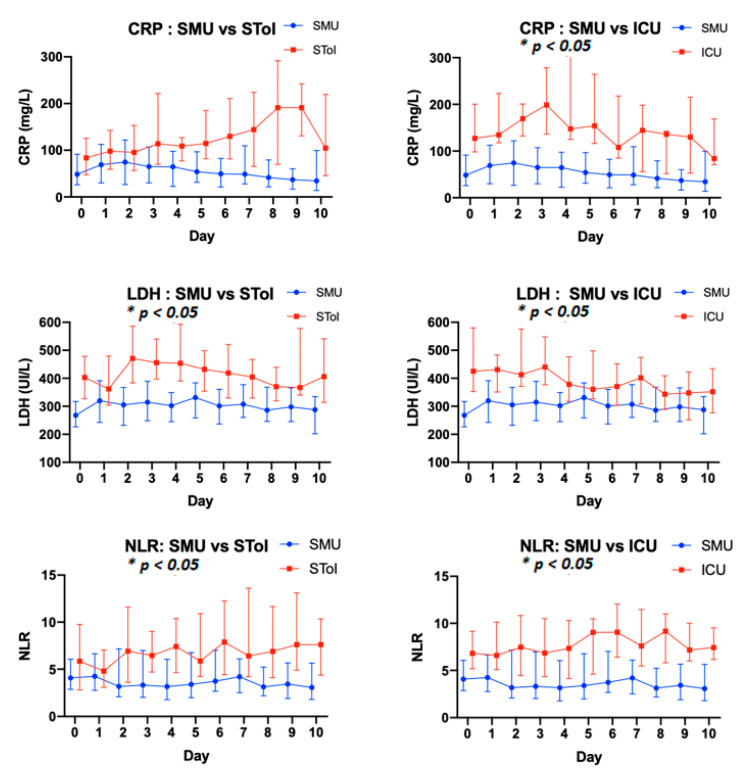
Kinetic following of biological parameters in training cohort (media and interquartile). Left column: Standard Medical Unit Patients vs. Standard to Intensive Care Patients groups. Right column: Standard Medical Unit Patients vs Intensive Care Units Patients. NLR: neutrophil–lymphocyte ratio, CRP: C-reactive protein, LDH: lactate dehydrogenase. Data were expressed as mean and range. Statistical analysis was performed to compare the kinetics of biological parameters over time (Day 0 to Day 10) between groups of patients (see Statistical analysis). * *p* < 0.05 mean that there was a significant difference in the behavior of parameters.

Regarding patient vital signs, notable differences were observed in general kinetic biological parameters between groups (C-reactive protein, neutrophil–lymphocyte ratio, Albuminemia, lactate dehydrogenase, Fibrinogen) (Figure 2).

Linear mixed models were performed for biological parameters, showing significant differences between the SMU, SToI and ICU groups during the two first days of hospitalization (Table 6), for PREDICT score training. The two-day timeframe was chosen because it represents the first quartile of time in which the standard medical unit to intensive care unit switch occurred in the SToI group. Furthermore, this follow-up period had to be long enough to provide enough time for physicians to react to and manage their patients and resources.

Results comparing SMU group versus SToI groups report Odds Ratios (OR) for C-Reactive Protein, Neutrophil–Lymphocyte ratio, and Lactate dehydrogenase of 2987.5 (95% CI: 10.7–836,6, *p*: 0.005), 61.9 (95% CI:1.7–2192.3, *p*: 0.023), and 60.6 (95% CI:3.1–1,17, *p*: 0.007), respectively, showing an increase in those parameters during the two first days of hospitalization.

### 3.4. Clinical-Biological Score for Predicting Intensive Care Risk

Considering all previous results, the most pertinent parameters were chosen to develop a score that is able to help physicians anticipate their patients’ deterioration and prepare for their transfer to intensive care unit, thus improving resource management.

This score can be calculated at admission (day 0), and then at day one and day two of hospitalization. It can deal with missing data for kinetic biological follow-up. It has an all-or-none approach for each criterion. For example, if a patient has a body mass index superior to 30, the score user must add nine points; if the patient has a body mass index lower than 30, no points are added.

**Figure 3:**
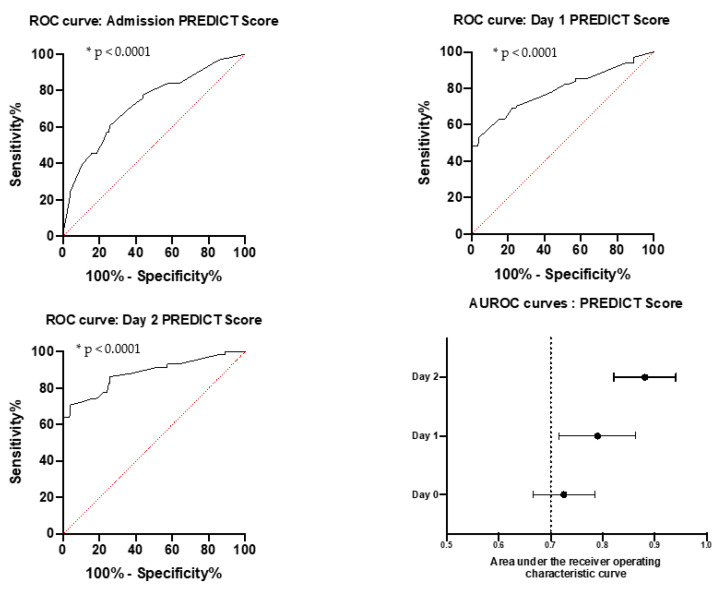
Receiver Operating Characteristic (ROC) curves for PREDICT score on admission in Intensive Care Unit; Day 0, day 1, and day 2 of hospitalization, and area under ROC curve repartition. * p < 0.05.

**Figure 4:**
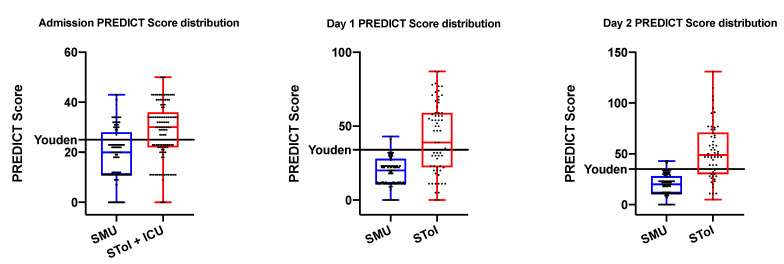
PREDICT score population construction repartition during the first two days of hospitalization, with maximum Youden index value (Cut-off). SMU: standard medical unit. SToi: need intensive care unit (ICU).

Three optimal thresholds were determined by maximum Youden index calculated on all points of the receiver operating characteristic curve. ROC curves are represented in Figure 3; they both have area under curve superior to 0.7 and they are all statistically significant, with a *p*-value inferior to 0.0001. Population division based on the PREDICT score is shown in Figure 4.

If a patient has a PREDICT score superior to the cut-off (Day 0: 25, Day 1: 34, Day 2: 35), no matter the day of calculation, he has an important risk of needing intensive care during hospitalization (Table 7).

## 4. Discussion

The major point that emerges from the present study is that the PREDICT score is useful to screen the COVID-19 hospitalized patients to locate those who need to be transferred to an intensive care unit. The population of this study is comparable to previous studies for baseline parameters [22,23] (gender, age, etc.). Furthermore, comorbidities like hypertension (defined using recent guidelines [24]), diabetes, and dyslipidemia showed similar prevalence rates, as reported by previous publications [25,26].

For example, the prevalence of obesity found in the SMU, SToI and ICU groups was 22.9%, 22.1%, and 49%, respectively. To simplify, we divided body mass index into only four categories (underweight, normal weight, overweight and obesity), without separating by obesity levels. Body mass index ≥ 30 was found to be a strong positive independent risk factor between our baseline population (SMU group) and ICU risk population. This very important proportion of patients with a body mass index ≥ 30 in need of ICU care has already been reported [21]. However, few significant differences between groups were found regarding other comorbidities, in contrast to what has been reported by other studies [27]. We believe that this is because the population included in our study already has a degree of disease severity, as it is composed of patients with a form of COVID-19 severe enough to warrant hospitalization. Thus, the comparison in our study is not with the general population, as has been in other studies, but with a population of hospitalized patients, whose baseline characteristics probably involve a higher degree of comorbidity.

Furthermore, a second crucial threshold was highlighted in this study. Age < 75 years, ages inferior to 75 years, were more likely to be admitted to intensive care unit by a statistically significant Odds Ratio (OR) after comparing the SMU group to the SToI and ICU groups. Such an Odds Ratio was previously found in the French national database [4] (Table 2, which allowed us to calculate this Odds Ratio to 5.6 (95% CI: [5.2–9.9]; *p* < 0.0001). This observation is surprising; however, two explanations could be proposed. First of all, the attack rate of SARS CoV-2 created a patient flow that surpassed our health care system’s capacity, imposing the need for a war-like medical triage system, in which the limited number of beds in intensive care units were assigned to patients that had the most chance of survival. Further, frail patients were admitted in a serious state, perhaps because of the hypoxic happiness phenomenon [28]

The main strength of our study is the kinetic follow-up of biological parameters. The study included common biological parameters, as we aimed to build a very user-friendly score. Further, it showed trends in accordance with previous analysis reported in the literature [29]. The comparison of kinetic follow-up during the two first days of hospitalization, with significative differences in C-reactive protein, lactate dehydrogenase and neutrophil–lymphocyte ratio, allowed us to apply our score during those first days. In clinical practice, this would allow physicians a comfortable time in which to evaluate the patient’s clinical course and react if necessary.

In Predict score at Day 0, the proportion of patients in the SMU groups who simultaneously reached the next three criteria: Age < 75 years and SpO_2_ ≤ 95% and respiratory rate ≥ 23 breaths/min was 9.7% in the training cohort and 9.1% in the validation cohort.

In our hospital, patients needing only high-flow oxygen treatment are managed in standard medical monitoring units. Admission to the ICU is indicated if desaturation occurs despite maximal high-flow oxygen therapy or another organ failure appears (cardiac, neurologic, hepatic or renal).

**Figure 5:**
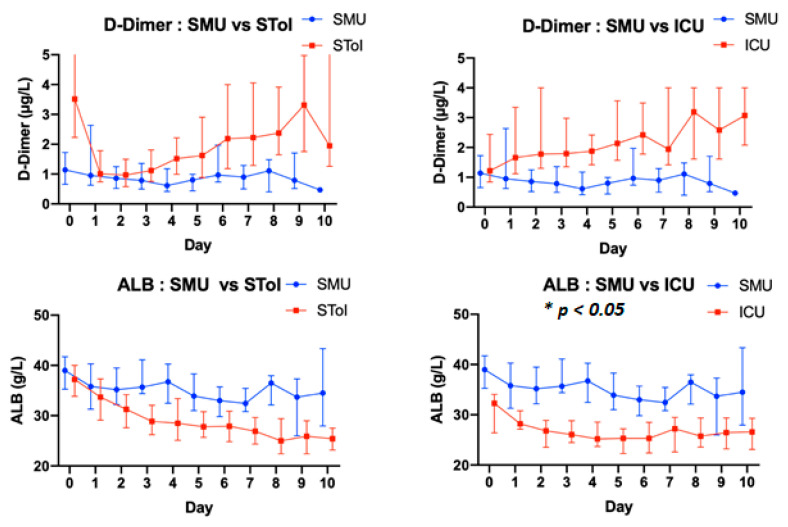
Kinetic follow-up of biological parameters. Left column: Standard Medical Units Patients vs. Standard to Intensive Care Patients. Right column: Standard Medical Units Patients vs. Intensive Care Units Patients. ALB: albuminemia.

Recent studies highlight the importance of biomarkers like D-Dimer, anticardiolipin IgG autoantibody, C-reactive protein, and interleukin-6 in the prediction of COVID-19 patients’ clinical decline [30,31]. Data regarding D-Dimer and albuminemia levels in our population show a trend towards higher D-dimer levels and lower albumin levels in both the STol and ICU groups (Figure 5). However, because our study was initiated at a time of crisis in France, there is a lack of data, which only allowed us to observe trends and prevented us from demonstrating statistical significance; this is a limitation to our study. Further, we were unable to demonstrate an association between the level of hypoalbuminemia during COVID-19 infection and risk of intensive care because of the confounding impact of dilution, despite previous studies showing its importance [32,33]. 

Moreover, a study proposing NEWS 2 as a tool for identifying patients at risk of requiring intensive care has been previously published [12], and we have compared its characteristics to those of our PREDICT score using a threshold of 5 for NEWS 2, as has been proposed (Table 8, Table 9, Table 10 and Table 11). NEWS 2 score is composed with the following variables: respiratory rate, oxygen saturation, need for supplemental oxygen, body temperature, blood pressure, heart rate and level of consciousness. In Anna Gidari publication [12], a threshold of 5 points in NEWS 2 is recommended for monitoring patients. The PREDICT score achieved a good sensitivity and a very good negative predictive value, which increased with each day it was performed, during the time allowed by the score (admission, day one and finally day two of hospitalization). A key point that emerges in both the training cohort and the validation cohort is the ability of the PREDICT score to correctly predict the need for transfer to intensive care of a patient already hospitalized for the management of COVID-19. In the SToI group of the training and validation cohorts, in patients in whom the PREDICT score was calculated only twice (those who went into intensive care after exactly 24 h of hospitalization had their PREDICT score calculated twice: at admission and day 1); at least one calculation was positive in 100% and 83.3%, respectively. Further, patients who needed transfer to intensive care unit after 48 h of hospitalization had a positive PREDICT score at least one time on admission, at day one or day two of hospitalization, with a 100% identification rate in both the training cohort and validation cohort.

Moreover, in patients from the SToI groups of the training and validation cohorts, regardless of the day they transferred from SMU into ICU, PREDICT score was positive at least once out of the two or three possible attempts (admission and/or day 1 in patients who switch after 24 h, and admission and/or day 1 and/or day 2 for those who switch after at least 2 days of hospitalization in SMU) in 83.8% and 86%, respectively. Contrastingly, NEWS2 correctly sorted only 56.9% of patients in the training cohort and 60.5% of patients in the validation population. However, the PREDICT score has limitations, as it correctly sorts only 44% and 47.1% of SMU patients in the training and validation cohorts, respectively.

Recent publications show strong works, multivariate analysis, multicentric analysis, but with different approaches to the PREDICT score, without considering biological parameters, which can precede clinical signs [34] or needing a computer to be calculated, which is clearly powerful but less easy to use [35]. Even if the approaches are different, the goal is the same: saving lives. The PREDICT score, as any other scoring system, is a tool; it could be used in parallel with other tools because it provides another point of view.

## 5. Conclusions

The PREDICT score uses simple parameters, is easy to use, and manually calculable. This study shows the potential of this score to anticipate the risk of intensive care necessity for COVID-19 patients hospitalized in standard medical units. However, it is a tool that must be employed by medical professionals in combination with their clinical analysis of the patient’s situation. The PREDICT score is powerful in identifying patients who require transfer from SMU to ICU, but less able to identify patients who need to be admitted to ICU in few hours; in such cases, the clinical sense of physicians is clearly dominant, and other tools, such as lactate values, could be employed. Moreover, the PREDICT Score classifies over 50% of patients hospitalized in SMU who will never need ICU care as in a risk category. Despite its imperfections, the PREDICT score correctly identifies patients who are at risk of needing intensive care.

## Figures and Tables

**Table 1:** Population’s characteristics (demographics data, important timelines, initial vital sign, income data, comorbidities, outcome data); comparison between groups for training cohort. Parameters with a *p*-value < 0.05 have significative differences between groups compared. * *p* < 0.05; ** *p* < 0.01; *** *p* < 0.001.

	All (*n* = 292)	SMU Group ^†^ (*n* = 175)	ICU Group ^†^ (*n* = 49)	SToI Group ^†^ (*n* = 68)	SMU vs SToI (*p*-Value)	SMU vs ICU (*p*-Value)	SToI vs ICU (*p*-Value)
**Demographics characteristics**							
Age. years. median [IQR]	68 [57–81]	74 [59–85]	62 [55–70]	67 [57–76]	** 0.004	*** <0.001	0.090
Age ≥ 75 years (%)	39.0	49.1	16.3	29.4	** 0.005	*** <0.001	0.102
Medically assisted nursing home	12	19.4	0.0	1.5	*** <0.001	** 0.001	1
Gender Male *(*%)	63.7	57.7	71.4	73.5	* 0.023	0.082	0.801
**Timeline (day)**							
Time between first symptoms and hospitalization. median [IQR]	5 [3–8]	5 [3–8]	7 [5–10]	5 [3–7]	0.931	** 0.004	** 0.004
Time between SMU and ICU. median [IQR]				4 [2–5]			
Time in SMU. median [IQR]		10 [7–14]					
Time in ICU. median [IQR]			22 [9–34]	8 [5–22]			
**Initial clinical characteristics**							
**Body Mass Index (kg/m^2^)**							
<18.5 (%)	1.4	1.7	0.0	1.5	0.44	*** <0.001	** 0.009
18.5–24.99 (%)	49.7	56.6	26.5	48.5			
25–29.99 (%)	21.9	18.9	24.5	27.9			
≥30 (%)	27.1	22.9	49.0	22.1			
**Comorbidities**							
Diabete (%)	34.5	32.2	43.8	33.8	0.807	0.136	0.278
Hypertension *(*%)	54.8	52.3	58.3	58.8	0.360	0.458	0.958
Cardio-Vascular diseases (%)	25.9	26.4	31.3	20.6	0.344	0.508	0.192
Dyslipidemia *(*%)	18.3	13.2	25	26.5	* 0.014	* 0.047	0.859
Chronic obstrucitve pulmonary disease (%)	6.2	5.2	6.3	8.8	0.372	0.725	0.734
Asthma *(*%)	7.2	8	10.4	2.9	0.248	0.569	0.124
Tobacco (%)	20	14.4	27.1	29.4	** 0.007	* 0.038	0.784
Active cancer (%)	7.6	9.2	6.3	4.4	0.214	0.771	0.690
Remission cancer (%)	4.5	5.2	4.2	2.9	0.733	1	1
Kidney disease (%)	6.9	6.9	6.3	7.4	1	1	1
**Symptoms on admission**							
Dyspnea (%)	52.9	37.4	95.9	61.8	** 0.001	*** <0.001	*** <0.001
Fever (%)	74.6	70.7	75.5	83.8	* 0.035	0.508	0.264
Cough (%)	55,0	51.7	51.0	66.2	* 0.042	0.931	0.099
Ageusia—Anosmia (%)	16.2	14.9	10.2	23.5	0.113	0.397	0.064
Diarrhea (%)	19.2	16.7	16.3	27.9	* 0.048	0.955	0.141
**Initial Vital signs**							
Heart rate. median [IQR]	90 [79–101]	89 [78–100]	94 [81–102]	90 [79–102]	0.344	0.126	0.511
Respiratory rate. median [IQR]	24 [19–28]	22 [18–26]	30 [25–35]	24 [20–30]	* 0.021	*** <0.001	*** <0.001
Systolic blood pressure. median [IQR]	132 [120–150]	133 [120–150]	126 [119–143]	130 [114–145]	0.23	0.132	0.678
Distolic blood pressure. median [IQR]	74 [63–83]	74 [64–82]	70 [61–83]	75 [63–87]	0.677	0.291	0.261
Temperature. median [IQR]	37.4 [36.8–38.3]	37.1 [36.7–38]	38.1 [37.1–38.8]	37.9 [37–38.5]	* 0.04	*** <0.001	0.265
Oxygen saturation (Sp O2) median [IQR]	95 [93–97]	96 [93–97]	94 [89–95]	95 [93–96]	* 0.036	*** <0.001	*** <0.001
**NEWS-2. median [IQR]**	5 [2–7]	4 [2–5]	7 [6–8]	5 [3–7]	** 0.002	*** <0.001	*** <0.001
Low risk (%)	48.1	61,0	5	43.1	** 0.002	*** <0.001	*** <0.001
Medium risk (%)	28	27.7	32.5	26.2			
High risk (%)	23.9	11.3	62.5	30.8			

Continuous variables were expressed as medians with interquartile ranges (IQRs) compared using Mann–Whitney U tests. Categorical variables were expressed as percentages (%) and compared using Chi-square test or Fisher’s exact test, as appropriate. ^†^ SMU group: patients only admitted to Standard Medical Unit, ICU group: patients directly admitted to Intensive Care Unit, SToI group: patients transferred from standard medical unit to intensive care unit. * *p* < 0.01: ** *p* < 0.01; *** *p* < 0.001

**Table 2:** Population’s characteristics (demographic data, important timelines, initial vital sign, income data, comorbidities, outcome data); comparison between groups for training cohort. Parameters with a *p*-value < 0.05 have significative differences between groups compared. ^†††^ Acute respiratory failure (ARF) was defined as Respiratory rate > 20 (or accessory muscle use for ventilation), and hypoxemia (PaO_2_ less than 60 mm Hg on breathing room air). ^††††^ Acute respiratory distress syndrome (ARDS): Acute respiratory failure not explained by cardiac failure or fluid overload with bilateral lung opacities on chest imaging and PaO_2_/FiO_2_ < 300 with positive end-expiratory pressure > 5 cm H_2_O. ^††^ Radiological lung damage severity was defined as Minor when patients had 3 compromised sites, with 3 lobules affected on each site (maximum 9 lobules), Intermediary, when patients had a minimum of 10 lobules affected, but less than 50% of total segmental volume, and Severe, when more than 50% of total segmental volume was affected. ^†^ SMU group: patients only admitted to Standard Medical Unit, ICU group: patients directly admitted to Intensive Care Unit, SToI group: patients transfered from Standard medical unit to Intensive care unit. * *p* < 0.05; ** *p* < 0.01; *** *p* < 0.001.

	All (*n* = 292)	SMU Group ^†^ (*n* = 175)	ICU Group ^†^ (*n* = 49)	SToI Group ^†^ (*n* = 68)	SMU vs SToI (*p*-Value)	SMU vs ICU (*p*-Value)	SToI vs ICU (*p*-Value)
**Initial O2 needed**							
Yes (%)	36.5	35.8	73.8	42.6	** 0.008	*** <0.001	** 0.001
Volume. median [IQR]	4 [3–9]	3 [2–5]	12 [5–15]	3 [2–6]	0.443	*** <0.001	*** <0.001
**Computed tomography (CT) low dose COVID-19**							
Yes (%)	94.2	98.3	75.5	97.1	0,622	*** <0.001	*** <0.001
^††^ Lung damages							
Absence (%)	6.9	10.4	0.0	1.6	*** <0.001	*** <0.001	*** <0.001
Minor (%)	22.5	29.9	2.9	14.1			
Intermediary (%)	39.3	42.7	11.8	45.3			
Severe (%)	31.4	17.1	85.3	39.1			
**Outcomes**							
Pulmonary embolism (%)	4.8	2.3	8.2	8.8	* 0.031	0.071	1
Cerbebral strocke (%)	1.7	0.6	6.1	1.5	0.482	* 0.034	0.307
Deep vein thrombosis (%)	7.2	1.7	22.4	10.3	* 0.06	*** <0.001	0.072
Total vascular insident (%)	12.7	4	32.7	20.6	*** <0.001	*** <0.001	0.140
Azithomycin (%)	91.4	94.3	79.6	92.6	0.767	** 0.003	* 0.037
Hydroxychloroquine (%)	56.2	49.1	61.2	70.6	** 0.003	0.135	0.289
^†††^ Acute repiratory failure (%)	47.3	12.6	100	98.5	*** <0.001	*** <0.001	1
^††††^ Acute respiratory distress syndrome [ARDS] (%)	37.7	5.1	98	77.9	*** <0.001	*** <0.001	** 0.002
Death (%)	16.8	14.3	20.4	20.6	0.230	0.297	0.981
**Maximum O2 help**							
**High-concentration mask**							
Yes (%)	9.6	0	16.3	30.9	*** <0.001	*** <0.001	
O2 Volume (L/min). Median [IQR]	30 [15–50]	NA	40 [28–50]	30 [15–50]	*** <0.001	*** <0.001	0.518
**Oro-tracheal intubation**							
Yes (%)	25	0	79.6	50	*** <0.001	*** <0.001	** 0.001

**Table 3:** Population’s characteristics (demographics data, important timelines, initial vital sign, income data, comorbidities, outcome data); comparison between groups for validation cohort. Parameters with a *p*-value < 0.05 have significative differences between groups compared. * *p* < 0.05; ** *p* < 0.01; *** *p* < 0.001.

	All (*n* = 140)	SMU Group ^†^ (*n* = 87)	ICU Group ^†^ (*n* = 10)	SToI Group ^†^ (*n* = 43)	SMU vs SToI (*p*-Value)	SMU vs ICU (*p*-Value)	SToI vs ICU (*p*-Value)
Demographics characteristics							
Age. years. median [IQR]	71 [61–81]	75 [62–85]	67 [59–74]	67 [59–72]	** 0.001	0.112	0.641
Age ≥ 75 years (%)	39.3	52.9	20.0	16.3	*** <0.001	*** <0.001	1
Medically assisted nursing home	7.9	11.5	10.0	0.0	* 0.03	* 0.043	0.189
Gender Male (%)	61.4	55.2	80	69.8	0.110	0.154	0.706
Timeline (day)							
Time between first symptoms and hospitalisation. median [IQR]	5 [3–7]	5 [3–7]	7 [2–13]	5 [3–7]	0.931	0.177	0.231
Time between SMU and ICU. median [IQR]				4 [2–6]			
Time in SMU. median [IQR]		8 [6–12]					
Time in ICU. median [IQR]			11 [6–17]	7 [3–20]			
Initial clinical characteristics							
**Body Mass Index (kg/m^2^)**							
<18.5 (%)	0,0	0.0	0.0	0.0	** 0.001	** 0.005	0.286
18.5–24.99 (%)	52.9	63.2	50.0	32.6			
25–29.99 (%)	20.0	18.4	30.0	20.9			
≥30 (%)	27.1	18.4	20.0	46.5			
Comorbidities							
Diabete (%)	42.1	35.6	50	53.5	0.059	0.174	1
Hypertension (%)	60	56.3	60	67.4	0.183	0.492	0.719
Cardio-Vascular diseases (%)	26.4	27.6	20	25.6	0.808	1	1
Dyslipidemia (%)	13.6	8	20	23.3	* 0.016	* 0.038	1
Chronic obstrucitve pulmonary disease (%)	8.6	8	0.0	11.6	0.530	0.616	0.570
Asthma (%)	5.0	2.3	10	9.3	0.092	0.134	1
Tobacco (%)	22.9	18.4	30	30.2	0.127	0.268	1
Active cancer (%)	13.6	12.6	0.0	18.6	0.365	0.370	0.327
Remission cancer (%)	7.1	6.9	0.0	9.3	0.729	0.874	0.473
Kidney disease (%)	8.6	10.3	10	4.7	0.336	0.472	0.345
Symptoms on admission							
Dyspnea (%)	65.7	60.9	90.0	69.8	0.323	0.146	0.258
Fever (%)	55.7	56.3	60.0	53.5	0.760	0.963	1
Cough (%)	37.1	40.2	20.0	34.9	0.556	0.462	0.471
Ageusia—Anosmia (%)	10.7	8.0	0.0	18.6	0.087	0.140	0.327
Diarrhea (%)	15.0	13.8	10.0	18.6	0.474	0.801	1
Initial Vital signs							
Heart rate. median [IQR]	88 [78–97]	84 [74–92]	99 [85–108]	91 [82–99]	** 0.008	* 0.033	0.301
Respiratory rate. median [IQR]	22 [18–28]	20 [18–25]	26 [24–31]	25 [20–28]	** 0.002	** 0.005	0.213
Systolic blood pressure. median [IQR]	130 [116–142]	130 [110–141]	132 [119–150]	130 [118–143]	0.577	0.525	0.724
Distolic blood pressure. median [IQR]	70 [61–79]	70 [60–79]	64 [50–84]	70 [63–80]	0.356	0.844	0.707
Temperature. median [IQR]	37.4 [36.8–38.3]	37 [36.6–38]	38 [37–39]	37.9 [36.9–38.5]	* 0.011	0.223	0.909
Oxygen saturation (Sp O2) median [IQR]	95 [92–96]	95 [93–97]	88 [80–95]	94 [92–96]	0.081	** 0.002	* 0.028
NEWS-2. median [IQR]	4 [2–6]	3 [1–5]	7 [5–9]	6 [4–7]	** 0.002	** 0.002	0.121
Low risk (%)	48.1	62.1	20	39.5	** 0.001	*** <0.001	0.331
Medium risk (%)	28	26.4	10.0	20.9			
Hight risk (%)	23.9	11.5	70.0	39.5			

Continuous variables were expressed as medians with interquartile ranges (IQRs) compared using Mann–Whitney U tests. Categorical variables were expressed as percentages (%) and compared using Chi-square test or Fisher’s exact test, as appropriate. ^†^ SMU group: patients only admitted to Standard Medical Unit, ICU group: patients directly admitted to Intensive Care Unit, SToI group: patients transfered from Standard medical unit to Intensive care unit.

**Table 4:** Population’s characteristics (demographics data, important timelines, initial vital sign, income data, comorbidities, outcome data); comparison between groups for validation cohort. Parameters with a *p*-value < 0.05 have significative differences between groups compared. ^†††^ Acute respiratory failure (ARF) was defined as Respiratory rate > 20 (or accessory muscle use for ventilation), and hypoxemia (PaO_2_ less than 60 mm Hg on breathing room air). ^††††^Acute respiratory distress syndrome (ARDS): Acute respiratory failure not explained by cardiac failure or fluid overload with bilateral lung opacities on chest imaging and PaO_2_/FiO_2_ < 300 with positive end-expiratory pressure > 5 cm H_2_O [20]. ^††^ Radiological lung damage severity was defined as Minor when patients had 3 compromised sites, with 3 lobules affected on each site (maximum 9 lobules), Intermediary, when patients had a minimum of 10 lobules affected, but less than 50% of total segmental volume, and Severe, when more than 50% of total segmental volume was affected. ^†^ SMU group: patients only admitted to Standard Medical Unit, ICU group: patients directly admitted to Intensive Care Unit, SToI group: patients transfered from Standard medical unit to Intensive care unit * *p* < 0.05; ** *p* < 0.01; *** *p* < 0.001.

	All (*n* = 140)	SMU Group ^†^ (*n* = 87)	ICU Group ^†^ (*n* = 10)	SToI Group ^†^ (*n* = 43)	SMU vs SToI (*p*-Value)	SMU vs ICU (*p*-Value)	SToI vs ICU (*p*-Value)
**Initial O2 needed**							
Yes (%)	34.3	32.2	30	39.5	0.407	0.714	0.725
Volume. median [IQR]	3 [2–5]	3 [2–5]	NA	3 [2–4]	0.885	* 0.019	
**Computed tomography (CT) low dose COVID-19**							
Yes *(%)*	96.4	95.4	88.9	100	0.301	0.140	0.173
Lung damages ^††^							
Absence (%)	5.7	8.4	0.0	2.4	0.1	0.070	0.496
Minor (%)	25.7	30.1	12.5	23.8			
Intermediary (%)	32.1	38.6	12.5	28.6			
Severe (%)	31.4	22.9	75.0	45.2			
**Outcomes**							
Pulmonary embolism (%)	2.9	1.1	10.0	4.7	0.254	0.093	0.473
Cerbebral strocke (%)	0	0	0.0	0.0			
Deep vein thrombosis (%)	0.7	0	0.0	2.3	0.331	0.379	1
Total vascular insident (%)	2.9	1.1	10.0	7	0.105	0.232	1
Azithomycin (%)	42.9	96.6	70.0	79.1	** 0.002	** 0.001	0.677
Hydroxychloroquine (%)	89.3	44.8	10.0	46.5	0.856	0.096	0.069
Acute repiratory failure ^†††^ (%)	50.7	23	90.0	97.7	*** <0.001	*** <0.001	1
Acute respiratory distress syndrome [ARDS] ^††††^ (%)	40.7	9.2	90.0	93	*** <0.001	*** <0.001	*1*
Death (%)	13	9.2	20.0	26.2	* 0.011	* 0.026	1
**Maximum O2 help**							
**High-concentration mask**							
Yes (%)	19.3	10.3		41.9	** 0.004	** 0.006	0.345
O2 Volume (l /min). median [IQR]	40 [28–50]	15 [15–25]	NA	45 [35–50]	*** <0.001		
**Oro-tracheal intubation**							
Yes (%)	20.7	0	80.0	48.8	*** <0.001	*** <0.001	*0.091*

**Table 5:** NEWS (National Early Warning Score) 2 scoring system calculation and interpretation.

**Physiological Parameter**	**Score**						
	+3	+2	+1	0	+1	+2	+3
Respiration rate (per min ute)	≤8		9–11	12–20		21–24	≥25
SpO_2_ scale 1 (%) *	≤91	92–93	94–95	≥96			
SpO_2_ scale 2 (%) *	≤83	84–85	86-87	88–92 ≥93 on air	93–94 on oxygen	95–96 on oxygen	≥97 on oxygen
Air or oxygen?		Oxygen		Air			
Systolic blood pressure (mmHg)	≤90	91–100	101–110	111–219			≥220
Heart rate (per minute)	≤40		41–50	51–90	91–110	111–130	≥131
Consciousness				Alert			New-onset confusion (or disorientation/agitation)
Temperature (°C)	≤35.0		35.1–36.0	36.1–38.0	38.1–39.0	≥39.1	
NEWS2 interpretation	Aggregate score = 0–4: Low clinical risk Aggregate score = 5–6: Medium clinical risk Aggregate score = 7 or above: High clinical risk						

* Oxygen saturation (SpO_2_) Scale 1: SpO_2_ on room air or supplemental O_2_ if patient has no hypercapnic respiratory failure. SpO_2_ Scale 2: If patient has hypercapnic respiratory failure.

**Table 6:** Statistical multivariate analysis of PREDICT score parameters during the first two days of hospitalization in standard medical unit (SMU). The two highest severity groups: SToI (need transfer to intensive care unit (ICU)) are compared to the referential group (Standard Medical Unit (SMU)). Parameters with a *p*-value < 0.05 have significative differences between groups compared.

					Odd Ratio	Confidence Interval (95%)	*p*-Value
	**Day 0**		**Day 0**			**Day 0**	
**Admission parameters**	**SMU Group**		**SToI + ICU Groups**		**SMU Vs (SToI + ICU)**		
Age < 75 years	50.8%		76%		231.2	[8.1; 6,611.4]	** 0.001
Body Mass Index ≥ 30 kg/m²	22.9%		29.1%		96.4	[4.8; 1,928.1]	** 0.003
Respiratory rate ≥ 23 breaths/min	40%		64.1%		348.7	[10. ; 11,567.9]	** 0.001
Oxygen saturation ≤ 95% (room air)	46.3%		64.1%		244.6	[9.2; 6,490.1]	** 0.001
Neutrophil-to-Lymphocyte Ratio ≥ 4	51.8%		80.6%		36.9	[1.1; 1,258.9]	* 0.045
	**Day 1**		**Day 2**			**Day 1 and 2**	
**Following parameters**	**SMU Group**	**SToI Group**	**SMU Group**	**SToI Group**	**SMU Vs SToI**		
Neutrophil–lymphocyte Ratio ≥ 6	32.4%	41.7%	29.7%	60%	61.9	[1.7; 2,192.3]	* 0.023
C- Reactive protein ≥ 53 mg/L	61.3%	80%	65.8%	85.2%	2987.5	[10.7; 836,567.9]	** 0.005
Lactate Dehydrogenase ≥ 450 UI/L	15.5%	35.5%	6.3%	64%	60.6	[3.1; 1,174.4]	** 0.007

**Table 7:** PREDICT score calculation table for the transfer to intensive care unit (ICU). Calculate Day 0 score by a simple sum. Day 1 score is the sum of Day 0 score plus day 1 biological potential point plus following adjustment 1 or 2. Finally, Day 2 score is the sum of day 1 score plus day 2 biological potential point plus following adjustment 1 or 2.

PREDICT Score (Predicting risk factors for Early Determination of ICU Transfer)	Day in Standard Medical Unit		
*5 Criteria on admission*	**Day 0**	**Day 1**	**Day 2**
**1. Age < 75 years**	+11		
**2. Body Mass Index ≥ 30kg/m²**	+9		
**3. Respiratory Rate ≥ 23 breaths/min**	+12		
**4. Oxygen saturation (SpO_2_) ≤ 95% (room air)**	+11		
**5. Neutrophil–lymphocyte Ratio ≥ 6**	+7		
**PREDICT score for high risk of ICU transfer**	**Score ≥ 25/50**		
*Take score of THE previous Day and add the 5 next criteria*		*Day 0 score plus*	*Day 1 score plus*
**1. Neutrophil–lymphocyte Ratio (NLR) ≥ 4**		+8	+8
**2. C-Reactive protein (CRP) ≥ 53 mg/L**		+16	+16
**3. Lactate dehydrogenase (LDH) ≥ 450 UI/L**		+8	+8
**4.** * following adjustment 1: **At least one of those 3 parameters is over its cut-off**		+12	+12
**5.** ** following adjustment 2: **None of those 3 parameters is over its cut-off**		−6	−6
**PREDICT score for high risk of ICU transfer**		**Score ≥ 34/94**	**Score ≥ 35/138**

* Following adjustment 1: if a patient has almost one biological parameters (neutrophil–lymphocyte ratio, C-reactive protein, lactate dehydrogenase) over the threshold, add 12 points. ** Following adjustment 2: if a patient has no biological parameters (neutrophil–lymphocyte ratio, C-reactive protein, lactate dehydrogenase) over the threshold, subtract 6 points.

**Table 8:** PREDICT and NEWS2 score characteristic comparison for training cohort. Se: sensitivity; Sp: specificity; PPV: Positive Predictive Value; NPV: Negative Predictive Value.

Training Cohort					
**NEWS**	D0	Se	71.4%	Sp	61.0%
Threshold	5	PPV	54.7%	NPV	76.4%
**PREDICT**	D0	Se	60.7%	Sp	74.3%
Youden	25	PPV	61.2%	NPV	73.9%
	D1	Se	58.8%	Sp	65.7%
Youden	34	PPV	40.0%	NPV	80.4%
	D2	Se	70.7%	Sp	54.9%
Youden	35	PPV	34.2%	NPV	85.0%

**Table 9:** PREDICT and NEWS2 score characteristic comparison for validation cohort. Se: sensitivity; Sp: specificity; PPV: Positive Predictive Value; NPV: Negative Predictive Value.

Validation Cohort					
**NEWS**	D0	Se	79.1%	Sp	62.1%
Threshold	5	PPV	50.7%	NPV	85.7%
**PREDICT**	D0	Se	54.7%	Sp	80.5%
Youden	25	PPV	63.0%	NPV	74.5%
	D1	Se	51.2%	Sp	59.8%
Youden	34	PPV	38.6%	NPV	71.2%
	D2	Se	70.3%	Sp	48.3%
Youden	35	PPV	36.6	NPV	79.2%

**Table 10:** Percentages of patients that have at least one positive occurrence for PREDICT score before their switch day (calculated at day 0 for ICU group, day 0 and day 1 for patients who switch after 1 day of hospitalization in SToI group, day 0 and day 1 and day 2 for patients who switch after at least 2 days of hospitalization in SToI group). Line SMU represent the percentage of patient with a positive score at least one time (day 0 and day 1 and day 2) in SMU group.

Training Cohort						
Groups	PREDICT (% of Patients with at Least 1 Occurrence Positive before Switch)					NEWS (Admission)
Day of switch to ICU	Day 0	Day 1	Day 2	>Day 2	Total	Total
SToI		100.0%	100.0%	77.1%	83.8%	56.9%
ICU	77.6%				77.6%	95.0%
SMU					56.0%	35.4%

**Table 11:** Percentages of patients that have at least one positive occurrence for PREDICT score before their switch day (calculated at day 0 for ICU group, day 0 and day 1 for patients who switch after 1 day of hospitalization in SToI group, day 0 and day 1 and day 2 for patients who switch after at least 2 days of hospitalization in SToI group). Line SMU represent the percentage of patient with a positive score at least one time (day 0 and day 1 and day 2) in SMU group.

Validation Cohort						
Groups	PREDICT (% of Patients with at Least 1 Occurrence Positive before Switch)					NEWS (Admission)
Day of switch to ICU	Day 0	Day 1	Day 2	>Day 2	Total	Total
SToI		83.3%	100.0%	81.3%	86.0%	60.5%
ICU	50.0%				50.0%	80.0%
SMU					52.9%	37.9%

## Data Availability

Data can be provided on C2VN (Aix Marseille University data base).

## Supplementary Materials

The following are available online at https://www.mdpi.com/article/10.3390/biomedicines9050566/s1, Table S1. NEWS (National Early Warning Score) 2 scoring system calculation and interpretation.

## Abbreviations

ICU	Intensive Care Unit
SMU	Standard Medical Unit
STol	Standard to Intensive Care
PREDICT score	Predicting Risk factors for Early Determination of ICU Transfer
AP-HM	Assistance Publique des Hôpitaux de Marseille (Public Assistance Hospital of Marseille)
Na	natremia
CRP	C-reactive protein
FRT	ferritinemia
LDH	lactate dehydrogenase
CREAT	creatinine
BILI	total bilirubin
ASAT	aspartate aminotransferase
ALAT	alanine aminotransferase
LY	lymphocyte count
NEU	neutrophils cells count
NLR	neutrophil–lymphocyte ratio
WHO	World Health Organization
RT-PCR	real time polymerase chain reaction
SpO_2_	arterial oxygen saturation
ROC	receiver operating characteristic
RR	respiratory rate
T °C	temperature
IQR	interquartile range
