# Supplementary material for: Predict Score: A New Biological and Clinical Tool to Help Predict Risk of Intensive Care Transfer for COVID-19 Patients
Source: Biomedicines. 2021 May 18;9(5):566. doi: 10.3390/biomedicines9050566 (PMC8157884; doi:10.3390/biomedicines9050566)
Supplement: Supplementary file 1 [file biomedicines-09-00566-s001.zip › biomedicines-1181268-supplementary.pdf]

Supplementary Table 1. NEWS (National Early Warning Score) 2 scoring system calculation and interpretation

Physiological parameter	Score						
	+3	+2	+1	0	+1	+2	+3
Respiration rate (per min ute)	≤ 8		9-11	12-20		21-24	≥ 25
SpO_2 scale 1 (%)*	≤ 91	92-93	94-95	≥96			
SpO_2 scale 2 (%)*	≤ 83	84-85	86-87	88-92<br>≥93 on air	93-94<br>on oxygen	95-96<br>on oxygen	≥ 97<br>on oxygen
Air or oxygen ?		Oxygen		Air			
Systolic blood pressure (mmHg)	≤ 90	91-100	101-110	111-219			≥ 220
Heart rate (per minute)	≤ 40		41-50	51-90	91-110	111-130	≥ 131
Consciousness				Alert			New-onset confusion (or disorientation/agitation)
Temperature (°C)	≤ 35.0		35.1-36.0	36.1-38.0	38.1-39.0	≥ 39.1	
NEWS2 interpretation	Aggregate score = **0-4**: **Low** clinical risk<br>Aggregate score = **5-6**: **Medium** clinical risk<br>Aggregate score = **7 or above**: **High** clinical risk						

* SpO_2 Scale 1: SpO_2 on room air or supplemental O_2 if patient has no hypercapnic respiratory failure.
SpO_2 Scale 2: If patient has hypercapnic respiratory failure
